# Evaluation of Planned Subgroup Analysis in Protocols of Randomized Clinical Trials

**DOI:** 10.1001/jamanetworkopen.2021.31503

**Published:** 2021-10-27

**Authors:** Ala Taji Heravi, Dmitry Gryaznov, Stefan Schandelmaier, Benjamin Kasenda, Matthias Briel

**Affiliations:** 1 Department of Clinical Research, Basel Institute for Clinical Epidemiology and Biostatistics, University Hospital Basel and University of Basel, Basel, Switzerland; 2 Department of Health Research Methods, Evidence, and Impact, McMaster University, Hamilton, Canada; 3 Department of Medical Oncology, University Hospital Basel, Basel, Switzerland; 4 Research and Development, iOMEDICO, Freiburg, Germany

## Abstract

This cross-sectional study compares randomized clinical trial protocols to assess the prevalence and reporting quality of planned subgroup analyses over time.

## Introduction

Well-researched and methodologically sound study protocols are important for the credibility of randomized clinical trials (RCTs).^1^ This is true for the main analysis and subgroup analyses.^2^ A 2014 study^3^ of RCT protocols approved by ethics committees between 2000 and 2003 found that almost 30% of protocols specified at least 1 subgroup analysis. However, most of them lacked essential details, such as the definition of subgroup variables, scientific rationales, hypotheses, or a description of statistical methods. In the present study, we compared these findings with 2 more recent samples of RCT protocols approved in 2012 and 2016 to assess the prevalence and reporting quality of planned subgroup analyses over time. In addition, we determined the proportion of planned subgroup analyses based on molecular and genetic markers.

## Methods

This cross-sectional study follows the Strengthening the Reporting of Observational Studies in Epidemiology (STROBE) reporting guideline. Approval by the ethics committee of Northern West and Central Switzerland and informed consent were waived because the study did not involve patients or the public in the design, conduct, reporting, or dissemination plans of the research.

This study uses data from 3 retrospective cohorts of RCT protocols approved between 2000 and 2003,^3^ 2012, and 2016.^1^ The examined protocols were approved by research ethics committees in Switzerland, Germany, and Canada. They constitute random samples of all approved RCT protocols at participating ethics committees. Investigators trained in clinical research methods (MSc or PhD) recorded, independently and in duplicate, RCT characteristics and details about subgroup analyses.^1,3^ Disagreements were resolved by discussion and consensus. We descriptively summarized the characteristics of the 3 cohorts focusing on the planning of subgroup analyses in RCT protocols in November and December 2020; comparative statements are not inferential. The present study is 1 of 5 prespecified subprojects of the Adherence to SPIRIT Recommendations (ASPIRE) study.^1^

## Results

This study included 894 protocols approved between 2000 and 2003, 257 protocols approved in 2012, and 292 protocols approved in 2016. At all 3 time points, approximately one-third of RCT protocols included plans for at least 1 subgroup analysis (2000-2003: 252 [28.2%]; 2012: 93 [36.2%]; 2016: 96 [32.9%]) (Table 1). At each time point, RCT protocols planning subgroup analyses were more frequently industry sponsored, had a multicenter design, and had a larger sample size than RCT protocols without planned subgroups. Subgroup analyses were particularly frequent in protocols of oncology and cardiovascular RCTs. The number of subgroup analyses per study, although frequently not reported, likely increased over time (2000 to 2003: median, 3 [IQR, 1-6]; 2012: median, 6 [IQR, 4-23.5]; 2016: median, 6 [IQR, 3-13]) (Table 2). The most frequent subgroup defining variables used in 2012 and 2016 (not assessed in the oldest sample) were age (2012: 44 of 93 [47.3%]; 2016: 42 of 96 [43.7%]) and sex (2012: 37 of 93 [39.7%]; 2016: 38 or 96 [39.5%]). Molecular or genetic markers were subgroup-defining variables in 13 of 93 (14.0%) RCT protocols approved in 2012 and 16 of 96 (16.7%) RCT protocols approved in 2016. The reporting of subgroup-specific hypotheses increased over time (2000 to 2003: 17 of 252 protocols [6.7%]; 2012: 9 of 93 protocols [9.7%]; 2016: 16 of 96 protocols [16.7%]) as did the number of plans that included a hypothesis sufficiently detailed to anticipate a direction of effect (2000 to 2003: 10 of 252 protocols [4.0%]; 2012: 9 of 93 protocols [9.7%]; 2016: 16 of 96 protocols [14.7%]). At all 3 time points, approximately one-third of subgroup analysis plans specified a statistical test for interaction (2000 to 2003: 87 of 252 protocols [34.5%]; 2012: 31 of 93 protocols [33.3%]; 2016: 26 of 96 protocols [27.1%]).

**Table 1.** Characteristics of Included Randomized Clinical Trial Protocols

Trial characteristics	Trial approval, No. (%)								
	2000-2003			2012			2016		
	SGA		All trials (n = 894)	SGA		All trials (n = 257)	SGA		All trials (n = 292)
	Not planned ( 642 [71.8%])	Planned (252 [28.2%])		Not planned (164 [63.8%])	Planned (93 [36.2%])		Not planned (196 [67.1%])	Planned (96 [32.9%])	
Median (IQR), target sample size	200 (80-471)	521 (229-1030)	260 (100-610)	165 (71.5-432)	600 (354-1500)	300 (100-720)	164 (75-416)	303 (150-600)	199 (100-490)
Center status									
Multicenter	500 (77.9)	241 (95.6)	741 (82.9)	119 (72.6)	91 (97.8)	210 (81.7)	131 (66.8)	84 (87.5)	215 (73.6)
Single center	139 (21.7)	10 (4.0)	149 (16.7)	45 (27.4)	2 (2.2)	47 (18.3)	65 (33.2)	12 (12.5)	77 (26.4)
Unclear	3 (0.5)	1 (0.4)	4 (0.4)	0	0	0	0	0	0
Study design									
Parallel	592 (92.2)	244 (96.8)	836 (93.5)	145 (88.4)	86 (92.4)	231 (89.9)	172 (87.8)	95 (99.0)	267 (91.4)
Crossover	40 (6.2)	1 (0.4)	41 (4.6)	10 (6.1)	1 (1.1)	11 (4.3)	11 (5.6)	1 (1.0)	12 (4.1)
Factorial	9 (1.4)	6 (2.4)	15 (1.7)	3 (1.8)	4 (4.3)	7 (2.7)	6 (3.0)	0	6 (2.1)
Other	1 (0.2)	1 (0.4)	2 (0.2)	6 (3.7)	2 (2.2)	8(3.1)	7 (3.6)	0	7 (2.4)
Study intention									
Superiority	456 (71.0)	196 (77.8)	652 (72.9)	130 (79.3)	73 (78.5)	203 (79.0)	160 (81.6)	79 (82.3)	239 (81.8)
Non-inferiority	95 (14.8)	44 (17.5)	139 (15.5)	23 (14.0)	19 (20.4)	42 (16.3)	30 (15.3)	14 (14.6)	44 (15.1)
Unclear	91 (14.2)	12 (4.8)	103 (11.5)	11 (6.7)	1 (1.1)	12 (4.7)	6 (3.1)	3 (3.1)	9 (3.1)
Sponsorship									
Industry	356 (55.5)	195 (77.4)	551 (61.6)	69 (42.1)	69 (74.2)	138 (53.7)	73 (37.2)	57 (59.4)	130 (44.5)
Investigator	286 (44.5)	57 (22.6)	343 (38.4)	95 (57.9)	24 (25.8)	119 (46.3)	123 (62.8)	39 (40.6)	162 (55.5)
Clinical area									
Oncology	113 (17.6)	42 (16.3)	155 (17.3)	22 (13.4)	25 (26.9)	47 (18.3)	27 (13.8)	24 (25.0)	51 (17.5)
Cardiovascular	59 (9.2)	49 (19.5)	108 (12.1)	8 (4.9)	19 (20.4)	27 (10.5)	15 (7.7)	20 (20.8)	35 (12.0)
Infectious diseases	60 (9.3)	27 (10.8)	87 (9.7)	6 (3.7)	3 (3.2)	9 (3.5)	4 (2.0)	3 (3.1)	7 (2.4)
Surgery	75 (11.7)	18 (7.2)	93 (10.4)	27 (16.5)	10 (10.8)	37 (14.4)	21 (10.7)	10 (10.4)	31 (10.6)
Pediatrics	34 (5.3)	11 (4.4)	45 (5.0)	11 (6.7)	3 (3.2)	14 (5.4)	11 (5.6)	8 (8.3)	19 (6.5)
Other	301 (46.9)	105 (41.7)	406 (45.4)	90 (54.9)	33 (35.5)	123 (47.9)	118 (60.2)	31 (32.3)	149 (51.0)

Abbreviation: SGA, subgroup analysis.

**Table 2.** Characteristics of Subgroup Analyses in Randomized Clinical Trial Protocols That Planned at Least 1 Subgroup Analysis

Characteristics of subgroup analyses	Trial approval, No. (%)		
	2000-2003	2012	2016
No.	252	93	96
Hypothesis given	17 (6.7)	9 (9.7)	16 (16.7)
Direction of subgroup effect anticipated	10 (4.0)	9 (9.7)	14 (14.6)
Interaction test planned	87 (34.5)	31 (33.3)	26 (27.1)
Subgroup outcome variable explicitly mentioned	NA	68 (73.1)	71 (74.0)
Exploratory nature explicitly mentioned	NA	33 (35.5)	22 (22.9)
Subgroup analysis considered in sample size calculation	NA	8 (8.6)	12 (12.5)
Most frequent subgroup variables^a^			
Age	NA	44 (47.3)	42 (43.7)
Sex	NA	37 (39.7)	38 (39.5)
Race and/or ethnicity	NA	25 (26.8)	22 (22.9)
Region	NA	23 (24.7)	14 (14.5)
Molecular/genetic markers	NA	13 (14.0)	16 (16.7)
BMI	NA	8 (8.6)	4 (4.1)
Subgroup analyses			
Median (IQR)	3 (1-6)	6 (4-23.5)	6 (3-13)
Not reported	30 (11.9)	31 (33.3)	43 (44.8)

Abbreviations: BMI, body mass index (calculated as weight in kilograms divided by height in meters squared); NA, not available.

^a^ More than 1 category possible.

## Discussion

The proportion and characteristics of RCT protocols with planned subgroup analyses appeared stable over time. Although the increasing proportion of hypothesis-supported subgroup analyses is encouraging, basic scientific principles, such as researching prior knowledge, limiting the number of analyses, and using appropriate statistics, continue to be violated in the majority of RCT protocols with planned subgroups. This is remarkable given the abundance of methodological guidance available.^2,4^ Study limitations include the poor reporting of subgroup analysis plans in some trial protocols and the lack of access to statistical analysis plans developed in later phases of trials. Considering the increasing importance of subgroup analyses to inform precision medicine,^5,6^ investigators and regulators should pay more attention to the methodological quality of subgroup analysis plans.

## Notes

Supplement.
Nonauthor Collaborators
