# Supplementary material for: Evaluation of Planned Subgroup Analysis in Protocols of Randomized Clinical Trials
Source: JAMA Netw Open. 2021 Oct 27;4(10):e2131503. doi: 10.1001/jamanetworkopen.2021.31503 (PMC8552052; doi:10.1001/jamanetworkopen.2021.31503)
Supplement: Supplement. — Nonauthor Collaborators [file jamanetwopen-e2131503-s001.pdf]

Supplemental Online Content: Nonauthor Collaborators

*Indicates required information. Only first name, last name, and suffix will appear in PubMed.

*Group Name(s): Adherence to SPIRIT Recommendations Study Group							
***First Name and Middle Initial(s)**	***Last Name**	***Suffix (eg, Jr, III)**	**Academic Degrees**	**Institution**	**Location (city, state/province, country)**	**Role or Contribution to the ASPIRE project, eg, chair, principal investigator**	**Group (if more than 1 Group listed in the byline) and/or Subgroup (eg, Steering Committee)**
Belinda	von Niederhäusern		PhD	Roche Pharma AG	Grenzach-Wyhlen, Germany	Coordination and data collection	
Benjamin	Speich		PhD	Basel Institute for Clinical Epidemiology and Biostatistics, Department of Clinical Research, University of Basel and University Hospital and Oxford Clinical Trials Research Unit / Centre for Statistics in Medicine, Nuffield Department of Orthopaedics, Rheumatology and Musculoskeletal Sciences, University of Oxford	Basel, Switzerland and Oxford UK	Coordination and data collection	
Elena	Ojeda-Ruiz		MD, MSc	Basel Institute for Clinical Epidemiology and Biostatistics, Department of Clinical Research, University of Basel and University Hospital and Infanta Elena University Hospital, Preventive Medicine Department	Basel, Switzerland and Madrid, Spain	Data collection	
Anette	Blümle		PhD	Cochrane Hungary, Clinical Centre of the University of Pécs, Medical School, University of Pécs	Pécs, Hungary	Data collection	
Dominik	Mertz		MD, MSc	Department of Health Research Methods, Evidence, and Impact, McMaster University	Hamilton, Canada	Data collection	
Ayodele	Odutayo		MD, PhD	Oxford Clinical Trials Research Unit / Centre for Statistics in Medicine, Nuffield Department of Orthopaedics, Rheumatology and Musculoskeletal Sciences, University of Oxford and Department of Anesthesia, McMaster University	Oxford, UK and Hamilton Canada	Data collection	
Yuki	Tomonaga		PhD	Epidemiology, Biostatistics and Prevention Institute, University of Zurich	Zurich, Switzerland	Data collection	

Supplemental Online Content: Nonauthor Collaborators

*Indicates required information. Only first name, last name, and suffix will appear in PubMed.

*First Name and Middle Initial(s)	*Last Name	*Suffix (eg, Jr, III)	Academic Degrees	Institution	Location (city, state/province, country)	Role or Contribution to the ASPIRE project, eg, chair, principal investigator	Group (if more than 1 Group listed in the byline) and/or Subgroup (eg, Steering Committee)
Alain	Amstutz		MD	Basel Institute for Clinical Epidemiology and Biostatistics, Department of Clinical Research, University of Basel and University Hospital and Swiss Tropical and Public Health Institute, University of Basel, and Department of Infectious Diseases and Hospital Epidemiology, University Hospital Basel	Basel, Switzerland	Data collection	
Christiane	Pauli-Magnus		MD	Department of Clinical Research, Clinical Trial Unit, University Hospital Basel and University of Basel	Basel, Switzerland	Data collection	
Viktoria	Gloy		PhD	Basel Institute for Clinical Epidemiology and Biostatistics, Department of Clinical Research, University of Basel and University Hospital	Basel, Switzerland	Data collection	
Szimonetta	Lohner		MD, PhD	Cochrane Hungary, Clinical Centre of the University of Pécs, Medical School, University of Pécs	Pécs, Hungary	Data collection	
Karin	Bischoff		MSc	Institute for Evidence in Medicine, Medical Center – University of Freiburg, Faculty of Medicine, University of Freiburg and Cochrane Germany, Cochrane Germany Foundation	Freiburg, Germany	Data collection	
Katharina	Wollmann		MSc	Institute for Evidence in Medicine, Medical Center – University of Freiburg, Faculty of Medicine, University of Freiburg and Cochrane Germany, Cochrane Germany Foundation	Freiburg, Germany	Data collection	
Laura	Rehner		MSc	Institute for Evidence in Medicine, Medical Center – University of Freiburg, Faculty of Medicine, University of Freiburg and Department of Epidemiology and Community Health, Institute for Community Medicine, University Medicine Greifswald	Freiburg, Germany and Greifswald, Germany	Data collection	

Supplemental Online Content: Nonauthor Collaborators

*Indicates required information. Only first name, last name, and suffix will appear in PubMed.

*First Name and Middle Initial(s)	*Last Name	*Suffix (eg, Jr, III)	Academic Degrees	Institution	Location (city, state/province, country)	Role or Contribution to the ASPIRE project, eg, chair, principal investigator	Group (if more than 1 Group listed in the byline) and/or Subgroup (eg, Steering Committee)
Joerg J.	Meerpohl		MD, MSc	Institute for Evidence in Medicine, Medical Center – University of Freiburg, Faculty of Medicine, University of Freiburg and Cochrane Germany, Cochrane Germany Foundation	Freiburg, Germany	Data collection	
Alain	Nordmann		MD, MSc	Department of Clinical Research, Basel Institute for Clinical Epidemiology and Biostatistics, University Hospital Basel and University of Basel	Basel, Switzerland	Data collection	
Katharina	Klatte		MSc	Basel Institute for Clinical Epidemiology and Biostatistics, Department of Clinical Research, University of Basel and University Hospital	Basel, Switzerland	Data collection	
Nilabh	Ghosh		MSc	Basel Institute for Clinical Epidemiology and Biostatistics, Department of Clinical Research, University of Basel and University Hospital	Basel, Switzerland	Data collection	
Kimberly	McCord		PhD	Department of Clinical Research, Basel Institute for Clinical Epidemiology and Biostatistics, University Hospital Basel and University of Basel	Basel, Switzerland	Data collection	
Sirintip	Sricharoenchai		MD, MSc	Department of Clinical Research, Basel Institute for Clinical Epidemiology and Biostatistics, University Hospital Basel and University of Basel	Basel, Switzerland	Data collection	
Jason W	Busse		PhD	Department of Health Research Methods, Evidence, and Impact, McMaster University, Hamilton, Canada & Department of Anesthesia, McMaster University	Hamilton, Canada	Data collection	
Arnav	Agarwal		MD	Department of Medicine, University of Toronto and Department of Health Research Methods, Evidence, and Impact, McMaster University	Toronto, Ontario, Canada and Hamilton, Canada	Data collection	
Ramon	Saccilotto		MD, MSc	Department of Clinical Research, Basel Institute for Clinical Epidemiology and Biostatistics, University Hospital Basel and University of Basel	Basel, Switzerland	Development of web-tool for data extraction	

*Indicates required information. Only first name, last name, and suffix will appear in PubMed.

*First Name and Middle Initial(s)	*Last Name	*Suffix (eg, Jr, III)	Academic Degrees	Institution	Location (city, state/province, country)	Role or Contribution to the ASPIRE project, eg, chair, principal investigator	Group (if more than 1 Group listed in the byline) and/or Subgroup (eg, Steering Committee)
Matthias	Schwenkglenks		PhD	Epidemiology, Biostatistics and Prevention Institute, University of Zurich, Zurich, Switzerland & Institute of Pharmaceutical Medicine (ECPM), University of Basel	Basel, Switzerland	Data collection	
Giusi	Moffa		PhD	Department of Clinical Research, Basel Institute for Clinical Epidemiology and Biostatistics, University Hospital Basel and University of Basel and Department of Mathematics and Computer Science, University of Basel	Basel, Switzerland	Data collection	
Lars G	Hemkens		MD, MPH	Basel Institute for Clinical Epidemiology and Biostatistics, Department of Clinical Research, University of Basel and University Hospital; Meta-Research Innovation Center at Stanford (METRICS), Stanford University; and Meta-Research Innovation Center Berlin (METRIC-B), Berlin Institute of Health	Basel, Switzerland; Stanford, California, US; Berlin, Germany	Data collection	
Sally	Hopewell		PhD	Oxford Clinical Trials Research Unit / Centre for Statistics in Medicine, Nuffield Department of Orthopaedics, Rheumatology and Musculoskeletal Sciences, University of Oxford	Oxford, UK	Conception, design, and data collection	
Erik	von Elm		MD, MSc	Cochrane Switzerland, Centre for Primary Care and Public Health (Unisanté), University of Lausanne	Lausanne, Switzerland	Conception, design, and data collection	
Jacqueline	Wong		PhD	Department of Health Research Methods, Evidence, and Impact, McMaster University	Hamilton, Canada	Data collection	
Ngai	Chow		PhD	Department of Health Research Methods, Evidence, and Impact, McMaster University	Hamilton, Canada	Data collection	
Patrik	Jiho Hong		PhD	Department of Health Research Methods, Evidence, and Impact, McMaster University, Hamilton, Canada & Department of Anesthesiology and Pain Medicine, University of Toronto	Toronto, Ontario, Canada	Data collection	
